# Urinary Metabolic Signatures Detect Recurrences in Non-Muscle Invasive Bladder Cancer

**DOI:** 10.3390/cancers11070914

**Published:** 2019-06-29

**Authors:** Alba Loras, M. Carmen Martínez-Bisbal, Guillermo Quintás, Salvador Gil, Ramón Martínez-Máñez, José Luis Ruiz-Cerdá

**Affiliations:** 1 Unidad Mixta de Investigación en Nanomedicina y Sensores, Universitat Politècnica de València-Instituto de Investigación Sanitaria La Fe, 46026 Valencia, Spain; 2 Instituto Interuniversitario de Investigación de Reconocimiento Molecular y Desarrollo Tecnológico, Universitat Politècnica de València, Universitat de València, 46022 Valencia, Spain; 3 CIBER de Bioingeniería, Biomateriales y Nanomedicina (CIBER-BBN), 28029 Madrid, Spain; 4 Departamento de Química Física, Facultad de Químicas, Universitat de València, 46100 Burjassot, Spain; 5 Analytical Unit, Instituto de Investigación Sanitaria La Fe, 46026 Valencia, Spain; 6 Health & Biomedicine, Leitat Technological Center, 08225 Terrassa, Spain; 7 Departamento de Química Orgánica, Facultad de Químicas, Universitat de València, 46100 Burjassot, Spain; 8 Departamento de Química, Universitat Politècnica de València, 46022 Valencia, Spain; 9 Unidad Mixta UPV-CIPF de Investigación en Mecanismos de Enfermedades y Nanomedicina, Universitat Politècnica de València, Centro de Investigación Príncipe Felipe, 46012 Valencia, Spain; 10 Servicio de Urología, Hospital Universitario y Politécnico La Fe, 46026 Valencia, Spain; 11 Departamento de Cirugía, Facultad de Medicina y Odontología, Universitat de València, 46010 Valencia, Spain

**Keywords:** bladder cancer, recurrence prediction, biomarker, metabolite, metabolomics, metabolic pathways, nuclear magnetic resonance

## Abstract

Patients with non-muscle invasive bladder cancer (NMIBC) undergo lifelong monitoring based on repeated cystoscopy and urinary cytology due to the high recurrence rate of this tumor. Nevertheless, these techniques have some drawbacks, namely, low accuracy in detection of low-grade tumors, omission of pre-neoplastic lesions and carcinomas in situ (CIS), invasiveness, and high costs. This work aims to identify a urinary metabolomic signature of recurrence by proton Nuclear Magnetic Resonance (^1^H NMR) spectroscopy for the follow-up of NMIBC patients. To do this, changes in the urinary metabolome before and after transurethral resection (TUR) of tumors are analyzed and a Partial Least Square Discriminant Analysis (PLS-DA) model is developed. The usefulness of this discriminant model for the detection of tumor recurrences is assessed using a cohort of patients undergoing monitoring. The trajectories of the metabolomic profile in the follow-up period provide a negative predictive value of 92.7% in the sample classification. Pathway analyses show taurine, alanine, aspartate, glutamate, and phenylalanine perturbed metabolism associated with NMIBC. These results highlight the potential of ^1^H NMR metabolomics to detect bladder cancer (BC) recurrences through a non-invasive approach.

## 1. Introduction

Bladder cancer (BC) is the most common cancer of the urinary tract. In men, BC ranks 7th worldwide with over 330,000 new cases per year and it is among the top 10 most deadly cancers [1]. At diagnosis, two different entities based on tumor stage are considered: Muscle-invasive bladder cancer (MIBC) and non-muscle-invasive bladder cancer (NMIBC). MIBCs represent ~20% of diagnosed BC and include tumors with a stage ≥T2. MIBC patients have a bad prognosis (5-year survival <50%) and require aggressive management [2], involving radical cystectomy followed by cycles of adjuvant chemotherapy, radiotherapy, or immunotherapy. On the other hand, ~80% of diagnosed BCs are NMIBCs, a heterogeneous group of tumors including papillary tumors limited to the mucosa (Ta, 70%) or invading the lamina propria (T1, 20%) and flat high-grade lesions confined to the mucosa (carcinoma in situ (CIS), 10%) [3]. NMIBC patients undergo transurethral resection (TUR) for tumor staging and afterwards they can be treated with intravesical instillation with Bacillus Calmette-Guérin (BCG) or mytomycin. NMIBC patients show better clinical outcomes than MIBC patients (5-year survival ~90%) [2] but they present high recurrence rates (up to 70% in the first 5 years), requiring lifetime follow-up. Cystoscopy and urinary cytology are routinely used for BC diagnosis and surveillance. Nevertheless, both of them present limitations, including issues related to accuracy in the detection of low-grade tumors, omission of CIS and pre-neoplasic lesions, invasiveness, and high costs [4,5,6].

To date, the Food and Drug Administration (USA) has approved six urinary diagnostic tests based on cytogenetics or proteins for BC diagnosis and/or follow-up [7,8]. Unfortunately, none of these biomarkers can be considered a reliable alternative to cystoscopy and advances in this field are therefore of importance.

In recent years, the use of “omic” approaches and liquid biopsy for biomarker discovery have begun to gain importance [8,9,10,11,12]. Between them, metabolomics offers dynamic biochemical information that can be linked to the phenotype of the disease, supporting the use of metabolites and metabolic profiles as a source of biomarkers of disease [13,14]. Metabolites are the downstream product of gene expression and play a role in the regulation of upstream molecular processes, such as transcription and translation [15,16]. Consequently, in the context of cancer, metabolomics has emerged as a widely informative technique for profiling metabolic features associated with specific oncotypes, tumor progression, therapeutic responses, and other clinical aspects related to tumor biology [17]. The latest advances in mass spectrometry (MS) and nuclear magnetic resonance (NMR), as well as in data analysis strategies, have fostered the use of metabolomics in this area of research [13,18]. In the context of BC, NMR and MS studies have focused on the identification of biomarkers for BC diagnosis or monitoring using urine and blood [19,20,21,22,23,24]. These studies have obtained good sensitivities and specificities (≈80–100%) comparing tumor versus health samples [25] and have shown taurine, citrate, and amino acid altered metabolism, among others, linked with BC. Nonetheless, previous studies have mostly focused on BC diagnosis and the detection of tumor recurrence during surveillance of NMIBC is still largely unexplored [19].

Urine could be the most suitable biofluid to develop a non-invasive BC biomarker. Urine is in contact with BC tumors and so the biochemical composition of the urine samples might be modified by molecules released from BC cells. This hypothesis is supported by recent preliminary results obtained by liquid chromatography–MS [24] that suggest the use of urinary biomarkers not only for BC detection, but also for patient monitoring during surveillance.

Inspired by this idea, we present here the results obtained in the frame of a clinical study aiming at the identification of metabolomic signatures for the non-invasive follow-up of NMIBC patients and the detection of recurrence according to the changes in the urinary metabolome before and after tumor resection by using ^1^H NMR spectroscopy. A multivariate model was calculated for the discrimination between urine collected before (BC) and after surgery (control). This model performance was then validated in urines collected from a cohort of NMIBC patients undergoing monitoring after TUR. Results showed the ability of this model to classify urines before and after surgery and to detect metabolic changes that occurred in the follow-up, supporting the use of a metabolic-based discriminant model for the detection of tumor recurrence in NMIBC.

## 2. Results

### 2.1. Urinary Metabolomic Profile in BC Patients

**Figure 1:**
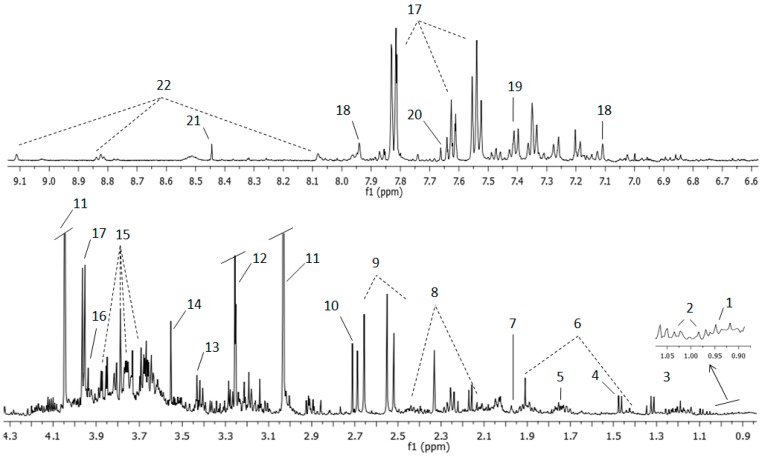
Representative ^1^H NMR spectrum and assignment of a urine sample from an NMIBC patient. Aromatic (top) and aliphatic region (bottom) are displayed. The spectral regions of water and urea have been removed from the figure. The intensity of the aromatic region is increased to show the resonances in this area, usually less intense than those observed in the aliphatic region. Peaks for the most intense resonances in the aliphatic region are shown truncated (11, 12, and 17) in order to improve the visualization of the other signals with lower intensity in this same area. Assigned metabolites: 1: Leucine, 2: Valine, 3: Lactate, 4: Alanine, 5: DSS (4,4-dimethyl-4-silapentane-1-sulfonic acid), 6: Lysine, 7: Acetic acid, 8: Glutamine, 9: Citrate, 10: Dimethylamine, 11: Creatinine, 12: Trimethylamine N-oxide, 13: Taurine, 14: Glycine, 15: Sucrose, 16: Creatine, 17: Hippuric acid, 18: Histidine, 19: Phenylalanine, 20: Pseudouridine, 21: Formic acid, 22: Trigonelline.

^1^H NMR water pre-saturation spectra were acquired for all urine samples with a good signal to noise ratio. Figure 1 displays a representative urine NMR spectrum from an NMIBC patient with the assignment of the main resonances. Table 1 summarizes the most relevant identified metabolites including amino acids (alanine, phenylalanine, glycine, lysine), benzenoids (hippuric acid), organic nitrogen compounds (trimethylamine-N-oxide), organic acids (lactate, citrate), creatinine, and urea.

### 2.2. Comparison of Metabolites Detected in Urine

**Figure 2:**
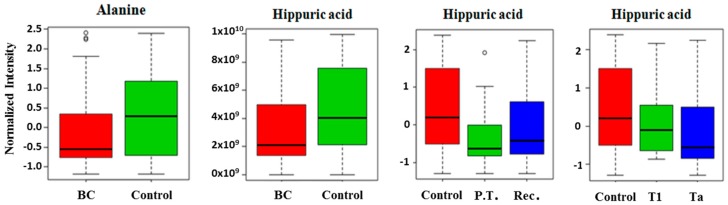
Box and whisker plots illustrating discrimination between: bladder cancer (BC) and control urines; control, primary tumor (P.T), and recurrences (Rec.); and differences among stages (Ta and T1) of BC and control urines.

Hippuric acid and alanine levels showed significant differences between BC and control groups (U-Mann–Whitney test, *p*-value < 0.05). Both metabolites presented lower intensities in BC samples. Hippuric acid also presented significant differences between primary tumors and recurrent tumors and between the Ta and T1 stages of tumors and control samples (Kruskal–Wallis test, *p*-value < 0.05) (Figure 2). Ta presented lower values for hippuric acid compared to control or T1 tumors and it seemed that primary tumors had lower hippuric acid values than recurrent or control urines.

### 2.3. Multivariate Analysis of the Urinary Metabolomic Profiles

An unsupervised Principal Component Analysis (PCA) was used to identify outliers or significant clustering of NMIBC urines (BC) and control samples in the score space. However, PCA did not reveal a sample clustering associated with malignancy. Then, a Partial Least Square Discriminant Analysis (PLS-DA) model was developed using tumor samples (coded as BC), and control samples (free of cancer), collected within 2–4 weeks after TUR (coded as CTRL). After model development, two external validation sets not used for model development, were considered in this study for the assessment of the predictive performance. The first validation set included BC and CTRL urines and the second validation set included these new BC and CTRL urines and incorporated a group of control urines (coded as MONITOR) collected during an active surveillance period of NMIBC patients (for more details see Material and Methods section). The split of non-BC urine samples in two sub-groups (i.e., CTRL and MONITOR) was done considering that, during monitoring, patients can be treated with BCG or chemotherapy, two potentially confounding factors that could affect sample classification. CTRL urines were collected from patients not subjected to any treatment.

**Figure 3:**
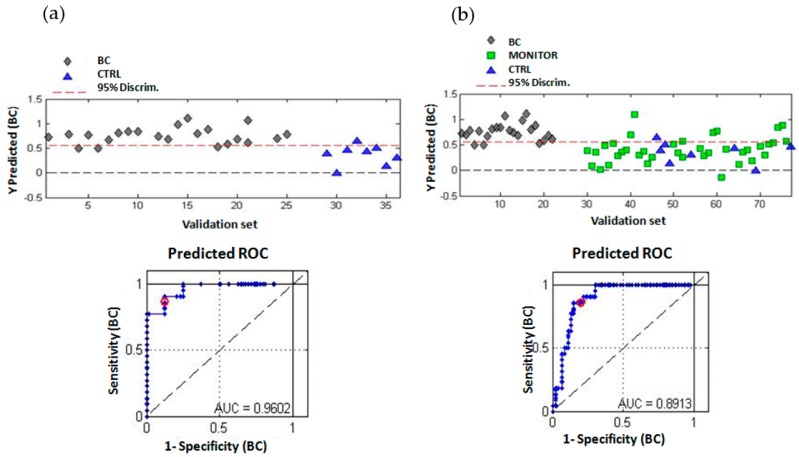
Discriminant analysis of BC, CTRL, and MONITOR samples. (**a**) Scores plot, PLS-DA predicted *y* values, and AUROC (area under receiver operating characteristic, ROC) for the first validation set (BC vs. CTRL); (**b**) Scores plot, PLS-DA predicted *y* values, and AUROC for the second validation set (BC vs. CTRL+MONITOR as control samples). Number of latent variables (LVs) = 3.

A permutation test (100 permutations) of results obtained by cross-validation showed a statistically significant *p*-value < 0.05 for the Wilcoxon test for a PLS-DA model build using three latent variables. PLS-DA predicted values for the validation sets shown in Figure 3 indicated a significant discrimination of BC and CTRL samples, also supported by figures of merit, which are summarized in Table 2. 

The PLS-DA model correctly classified 19/22 BC and 7/8 CTRL samples (see Table S1) providing a sensitivity of 86.4%, a specificity of 87.5%, and an area under the receiver operating characteristic curve (AUROC) = 0.96 with an accuracy (ACC) of 86.7% for BC and CTRL samples included in the validation set. When MONITOR samples were included in the validation set, the sensitivity value was maintained but the model provided a lower specificity. Nonetheless, AUROC was 0.89% and the negative predictive value (NPV) and negative likelihood ratio (NLR) were 92.7% and 0.17%, respectively.

**Figure 4:**
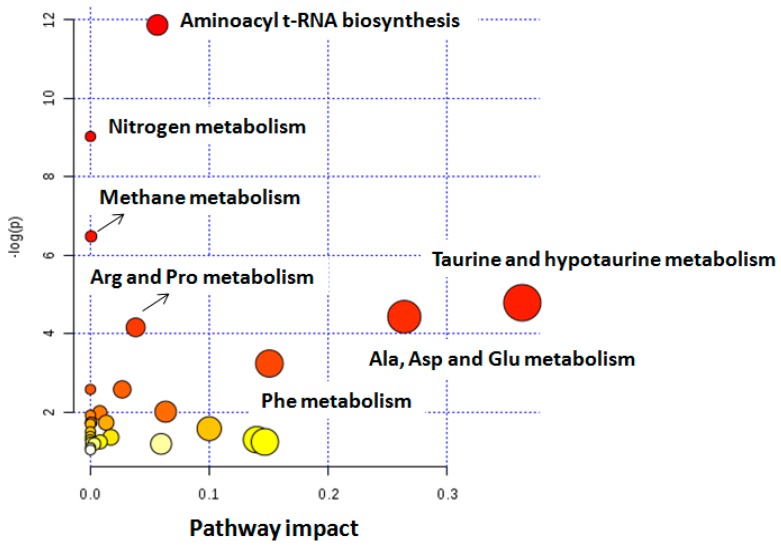
Analysis of altered metabolic pathways in BC. Note: the color and the size of each circle indicate its *p*-value and pathway impact value, respectively. Ala: alanine; Arg: arginine; Asp: aspartate; Glu: glutamine; Phe: phenylalanine; Pro: proline.

The most discriminant metabolites between BC and control samples in the PLS-DA, considering a variable importance in projection (VIP) score > 1 as threshold value, included valine, alanine, lysine, glutamine, citrate, dimethylamine, creatinine, trimethylamine N-oxide, taurine, sucrose, creatine, hippuric acid, histidine, phenylalanine, and trigonelline. The pathway analysis performed in MetaboAnalyst linked these metabolites with alterations in taurine and hypotaurine, alanine, aspartate, glutamate, and phenylalanine metabolic pathways, among others (*p*-value < 0.05) (see Table 3 and Figure 4).

### 2.4. Analysis of Changes in the Metabolic Profile during the Follow-Up Period of NMIBC Patients

**Figure 5:**
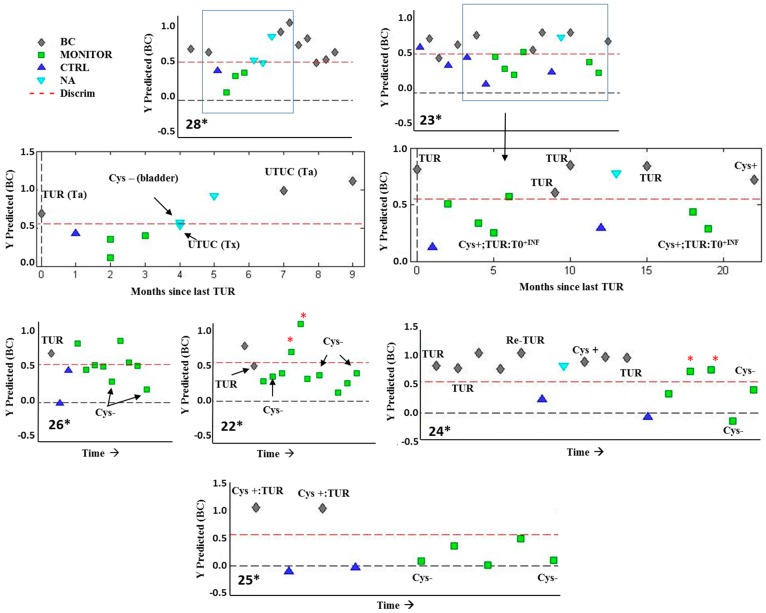
Analysis of longitudinal metabolic trajectories after transurethral resection (TUR) of BC. Predicted *y* Partial Least Square Discriminant Analysis (PLS-DA) values in six patients during the follow-up period. Note: (*) indicates a MONITOR sample showing an inconsistent trajectory; (Cys+ or Cys−) indicates a positive or negative cystoscopy, respectively; (T0) indicates tumor absence by PA evaluation; (Tx) indicates that the pathologist did not confirm the presence of a tumor; (INF) indicates cystitis; (UTUC) means upper tract urothelial carcinoma.

The PLS-DA model performance in the validation set including MONITOR samples correctly classified 19/22 BC and 37/46 as non-tumor (i.e., CTRL or MONITOR) samples (see Table S1). Out of the total patients (*n* = 28), these CTRL and MONITOR samples were collected from seven patients undergoing active follow-up who had different clinical evolutions. Longitudinal metabolic trajectories observed for these patients are shown in Figure 5, where results obtained from the analysis of urine samples without cystoscopic evaluation (NA) were included. The longitudinal trajectory of the metabolomic profile of these patients allowed the assessment of the utility of this PLS-DA model to detect tumor recurrences in urine during a surveillance period. For example, results depicted for patients 23*, 24*, 25*, and 28* in Figure 5 were in agreement with the results of cystoscopy and pathological anatomy (PA) after TUR. Moreover, in patients 23*, 24*, 25*, and 28*, a shift in the metabolomic profile from control to tumor was observed along with its detection by positive cystoscopy; this was confirmed by PA. The metabolic profile observed in the urine was restored to the control phenotype after tumor removal. In patients who did not develop many recurrences, the metabolic profile remained in a non-tumor phenotype after TUR during a monitoring period and tumor absence was confirmed through negative cystoscopies (see patients 22*, 26*, and 25* in Figure 5). It deserves to be mentioned that the observed metabolic profile was not affected by inflammatory processes (i.e., cystitis), since non-tumor urines collected during these circumstances were correctly classified as non-tumor urines (see patient 23*) (Figure 5).

## 3. Discussion

NMIBC patients require complex clinical management due to the high heterogeneity of tumors. Patients with equal PA diagnoses have completely different evolutions, indicating a unique biology for each tumor. Tumors represent dynamic entities that are continuously changing their molecular programs to adapt to the microenvironment conditions. That ability to adapt depends, in part, on metabolic reprogramming, a phenomenon recognized as an emerging hallmark of cancer [26]. Therefore, the analysis of metabolic changes produced in biological samples can offer opportunities for biomarker discovery.

We developed a PLS-DA model to discriminate between pre-TUR NMIBC (BC) and post-TUR (CTRL) samples that provided an elevated sensitivity (86.4%), specificity (80.9%), and NPV (92.7%). These results suggested that the metabolic profile could be a very useful, non-expensive, and non-invasive strategy in clinical practice to detect recurrences during monitoring. This observation was assessed by the analysis of metabolic longitudinal trajectories of PLS-DA predicted values in six patients with NMIBC undergoing active follow-up. The metabolic profile of patient 25* changed from BC toward the control phenotype after tumor removal by TUR in the two developed tumor episodes. Negative cystoscopies confirmed the absence of recurrences in agreement with the metabolic profile trajectories. A similar profile was observed in patients 26* and 22*, although in these cases, four MONITOR samples were misclassified. Further research is needed to assess the source of error in these cases (e.g., drugs, diet, treatment, tumor phenotype). The metabolic profile of patient 24* remained in a BC phenotype between the first complete TUR and a re-TUR performed five months later. The re-TUR was programmed due to the size of the tumor (5–7 cm) and the PA (Ta G3) of the previous tumor. During re-TUR, three tumors were found with a PA (T1 G2). The metabolic signature was in agreement with early detection of the recurrence. Patient 23* had a large number of recurrences after the diagnosis of the primary tumor. The classification of BC samples was in concordance with positive cystoscopy and the PA of tumor after TUR (see samples collected in the months 9, 10, and 22). The correct classification of CTRL and MONITOR samples was in concordance with the negative results of PA (T0) (see the results obtained for samples collected in months 5 and 19 in Figure 5). In these cases, the patient had been operated in response to tumor suspicion (positive cystoscopy). However, the diagnosis of the tumor specimen was cystitis. Remarkably, patient 28* had a recurrence in the bladder (see the results obtained for the sample collected in month 0 in Figure 5) and another in the upper tract (UTUC) with the same PA (see month 7). The metabolic signature detecting the UTUC development was confirmed by PA in the 7th month. Negative cystoscopies performed in months 4 and 7 showed the absence of tumors in the bladder epithelium. 

In general, the obtained results indicated that the metabolic profile could be useful in very diverse clinical situations linked with NMIBC. The profile proved to be highly dynamic and sensitive in the detection of bladder recurrences, not only in very early stages of their development, but also in the detection of recurrences in the upper urinary tract. This has relevance considering the fact that BC could be considered a pan-urothelial disease. Moreover, in some cases, the metabolic profile was able to detect incipient tumors not observed by cystoscopy at the time of the urine sample collection, but identified in the subsequent examination. From the clinical point of view, if these results were confirmed in further studies, it would enable personalized follow-up schemes for a better control of the disease during monitoring. Furthermore, the application of this metabolic profile in the detection of incomplete TUR would be essential, since this would provide useful information to select the best treatment for each patient; a strict control by urinary cytology or cystoscopy, a re-TUR, or a new regimen of chemotherapy or immunotherapy. By contrast, if the urinary signature indicated a control phenotype after TUR, a re-TUR could be avoided. Surgeries could be also prevented in those cases where cystoscopy confounds cystitis with tumors, but after TUR the PA shows tumor absence (i.e., T0). In brief, these data reinforce the idea that urinary metabolome reflects tumor biology and can be used to study tumor development. Nevertheless, although the obtained results are hopeful, a better understanding of how several intrinsic and extrinsic factors, such as the effect of chemotherapy or BCG, drugs, and inflammatory processes affecting the urinary metabolome, are essential to improve the robustness of metabolic tests so they can be implemented into clinical routine.

On the other hand, the knowledge which metabolites build upon in this profile is important to establish the link between altered metabolic pathways and the tumor phenotype, which indirectly reflects the events developed at the genomic and transcriptomic levels. A better knowledge of metabolic regulation would allow the detection of key metabolic enzymes that could be a target for the development of new BC therapies.

Among the metabolites identified as discriminants between BC and control urines (VIP > 1), we highlighted glutamine, glutamate, citrate, hippuric acid, and taurine. Changes in these metabolites have been described as relevant in several tumors (e.g., prostate, breast, ovarian) and their roles in cellular metabolism are quite well-known [27,28,29]. Furthermore, these metabolites have been detected as discriminant in our previous urinary study based on ^1^H NMR [24], so the results presented here validate and reinforce the importance of them in BC cell metabolism. Glutamine and glutamate have been described as two important metabolites in BC [19]. Cancer cells use glutamine as source of energy but also for nucleotide biosynthesis or the synthesis of other aminoacids [30]. The enzyme glutaminase converts glutamine to glutamate, which can be used by alanine aminotrasferase to produce intermediates of the TCA cycle or for the synthesis of glutathione, a tripeptide that acts as an important antioxidant in cells [31]. Glutamine uptake is also linked with the immune system, since several T cell metabolic processes require it [32]. Citrate is a key intermediate of the TCA cycle and has been closely related to an increase in fatty acid β-oxidation to support cancer cell proliferation. Although we did not find significantly different levels of citrate between tumor and non-tumor urines, low concentrations of this metabolite have been reported in bladder tumors, suggesting the role of β-oxidation as source of energy [23]. Additionally, alterations in taurine metabolism have been shown in NMIBC [33,34], which is in accordance with our results. The lower levels of hippuric acid found in our BC urines compared to control samples are in agreement with previous works [23,33]. However, we also identified differences in the levels of hippuric acid among urines collected from controls and patients with primary and recurrent tumors, suggesting differences in the metabolism of these types of tumors. Overall, the results presented here are supported by prior references [35] and in concordance with our previous results obtained by ultra performance liquid chromatography mass spectrometry (UPLC-MS) showing the perturbation of phenylalanine, arginine, proline, and tyrosine metabolic pathways, among others, in NMIBC [23]. Nevertheless, the study presented here using NMR-based metabolic urine profiling has advantages over MS that include minimal sample preparation, non-destructive analysis, higher reproducibility, and cost-effectiveness. 

## 4. Materials and Methods

### 4.1. Patient Selection and Study Design

This study was approved by the Ethics Committee for Biomedical Research of the Instituto de Investigación Sanitaria Hospital Universitario y Politécnico La Fe (Valencia, Spain) (approval number 2012/0186) and all patients gave written informed consent to participate. 28 BC patients (20 males and 8 females) were recruited in the urological service of the Hospital Universitario y Politécnico La Fe. Inclusion criteria for patient selection included being male or female from 20 to 90 years old, NMIBC diagnosed, transitional cell carcinoma, single or multiple tumors, and primary or recurrent bladder tumors. Exclusion criteria included patients with urinary catheter, invasive tumor (T2–T4), unique papilloma, or other types of bladder tumor diagnoses such as carcinoma in situ, squamous cell carcinoma, adenocarcinoma, small cell carcinoma, or sarcoma.

From these patients, 153 urine samples were collected between March 2012 and December 2016. Patients were included in a monthly monitoring group with urinary cytology, cystoscopy, and serial urine collection until recurrence. Urines were processed and stored at −80 °C by the Biobanco La Fe (PT13/0010/0026). According to the results of urinary cytology, cystoscopy, and pathological anatomy, urines were incorporated into the tumor (BC) or control group. If no cystoscopy was available at the time or after urine collection, urines were classified as “NA” (non-available cystoscopic evaluation). The tumor group, encoded as BC, included 70 urines collected from NMIBC patients when cystoscopy was positive and PA confirmed the presence of tumor and one month before positive cystoscopy with tumors ≥ 3 cm. All bladder tumors considered in this study were transitional cell carcinomas. The non-BC group comprised two subgroups: CTRL (*n* = 29) and MONITOR (*n* = 38). The CTRL group included urines collected from NMIBC patients within 2–4 weeks after TUR. The MONITOR group included urines collected during the monitoring period with negative PA (T0), urines with negative cytology at the time of collection, and urines collected during the monitoring period between negative cystoscopies. The division of control samples between the two groups was performed considering that MONITOR urines were collected during a period in which patients could be treated with BCG or chemotherapy. In contrary, CTRL urines were collected after surgery, when patients were not subjected yet to any treatment. Nevertheless, if patients underwent treatment during the follow-up period, MONITOR urines were collected before treatment administration and at least one week after previous treatment, so MONITOR urines were not affected by drugs. Table 4 summarizes clinical–pathological data of patients and samples included in the study.

### 4.2. Sample Preparation and ^1^H NMR Acquisition

Urine samples were thawed at room temperature and pre-processed following already published procedures [36]. Briefly, phosphate buffer (200 μL, 7.4 pH) was added to urine (500 μL). The mixture was homogenized, centrifuged at 10,000 rpm for 5 min at 5 °C, and transferred to a 5 mm NMR tube. For each urine sample, 1D ^1^H water pr-esaturation spectrum was acquired at 300 °K using a Bruker Avance II 500 MHz spectrometer (Bruker GmbH, Rheinstetten, Germany) with the following spectral acquisition parameters: Acquisition time—2.72 s; transients—128; spectral width—12 ppm (6000 Hz); relaxation delay—5 s. Moreover, 2D ^1^H–^13^C HSQC (heteronuclear single-quantum correlation) spectra were acquired in two representative samples to assess the assignments of the overlapped signals in 1D ^1^H NMR spectra.

### 4.3. ^1^H NMR Spectra Pre-Processing and Metabolite Assignment

After spectra acquisition, the free induction decay (FID) was Fourier transformed, phase and baseline corrected, and chemical shift referenced to 4,4-dimethyl-4-silapentane-1-sulfonic acid (DSS) at 0.0 ppm using MestReNova version 6.0.2 (Mestrelab Research SL, Santiago de Compostela, Spain). Metabolites were identified and assigned according to the published data [37,38,39,40] and NMR databases [41,42], considering a peak tolerance of ± 0.02 ppm. The spectra were binned into 0.003 ppm buckets using MestReNova. For the statistical analysis, the 0.8–4.5 and 6.5–9.0 ppm regions were considered, thus excluding the spectral regions where water and urea resonances appeared.

### 4.4. Data Analysis

The intensity of the assigned resonances was transferred to MetaboAnalyst 3.0 [43]. Intensities in each spectrum were normalized by the sum (1-norm) to avoid the contribution of urine dilution. Mean metabolite intensities were compared between groups. The U-Mann–Whitney test was used to identify significant differences between BC and control urines (CTRL and MONITOR). Kruskal–Wallis was used to evaluate differences among control (CTRL and MONITOR), primary tumors, and recurrence tumors and to compare between Ta, T1, and CTRL groups.

Multivariate statistical analysis was carried out using the PLS_Toolbox Solo 8.0 (Eigenvector Research Inc., Manson, WA, USA). Data pre-processing included first-derivative, row-wise normalization by the sum (1-norm) to avoid the contribution of urine dilution and autoscaling. PCA including all the samples (BC, CTRL, MONITOR, and NA) was carried out to detect potential outliers. Then, the data set was split into calibration and validation subsets. Samples from 24 patients (69 urines) were included in the calibration set (21 CTRL and 48 BC) and were used to calculate a PLS-DA model to discriminate between BC and CTRL samples (Table 4). The optimal number of latent variables (LVs = 3) was selected according to the root mean square error of cross-validation (RMSECV). A validation set including urines from 7 patients with different clinical evolutions was used to evaluate the predictive performance of this model to predict BC and CTRL urines and also urine changes during monitoring. The assessment of the predictive performance was performed in two data sets. The first included spectral data acquired from 22 BC and 8 CTRL urines. Then, follow-up samples (38 MONITOR) were included in the validation set to test the performance of the model to detect tumor recurrence during the surveillance period (see Table 4 and Table S2). NA samples (*n* = 16) were included in the validation for qualitative analysis of the trajectory of the PLS-DA predicted values in patients under surveillance.

A permutation test (100 permutations) was carried out to assess the statistical significance of PLS-DA figures of merit and the probability of a chance correlation using the Wilcoxon test. The relative importance of each metabolic feature in the PLS-DA model was determined using the VIP scores vector. Metabolites with characteristic resonances showing VIP scores > 1 in the PLS-DA model were selected to perform pathway and topology analysis using MetaboAnalyst 3.0 [43] using a global test and a relative betweenness centrality measure.

## 5. Conclusions

In summary, the present study shows, for the first time, a dynamic ^1^H NMR-based urinary metabolic profile associated with NMIBC that changes from a tumor to a control phenotype after tumor removal and returns to the malignant condition when a recurrence occurs. This fact highlights metabolomics as a tool for searching non-invasive biomarkers, which could be applied in clinical practice to improve the management of BC patients by: (1) Decreasing the performance of unnecessary cystoscopies during the follow-up period; (2) detecting lesions not visible by cystoscopy such as dysplasias, hyperplasias, and CIS and (3) detecting early recurrences, incomplete TUR, or UTUC.

## Figures and Tables

**Table 1:** Assignment of the main metabolites identified in 1D ^1^H NMR urine spectra.

Number Assignment	Metabolite	Group	Chemical Shift ppm
1	Leucine	CH_3_	0.94
2	Valine	γCH_3_	0.98
2	Valine	γCH_3_	1.03
3	Lactate	CH_3_	1.32
6	Lysine	γCH_2_	1.43
4	Alanine	βCH_3_	1.47
6	Lysine	βCH_2_	1.71
7	Acetic acid	CH_3_	1.97
6	Lysine	βCH_2_	1.91
8	Glutamine	βCH_2_	2.13
8	Glutamine	γCH_2_	2.44
9	Citrate	CH_2_	2.51
9	Citrate	CH_2_	2.65
10	Dimethylamine	CH_3_	2.71
11	Creatinine	CH_3_	3.03
13	Taurine	-CH_2_-NH_3_^+^	3.25
12	Trimethylamine N-oxide	CH_3_	3.29
13	Taurine	-CH_2_-SO_3_^-^	3.42
14	Glycine	αCH	3.55
15	Sucrose	C6′H_2_	3.81
15	Sucrose	C5′H	3.87
16	Creatine	CH_2_	3.92
17	Hippuric acid	αCH_2_	3.96
11	Creatinine	CH_2_	4.05
18	Histidine	CH	7.09
19	Phenylalanine	C2′6H	7.33
19	Phenylalanine	C3′5H	7.41
17	Hippuric acid	C3′5H	7.63
20	Pseudouridine	CH	7.66
17	Hippuric acid	C2′6H	7.82
18	Histidine	CH	7.93
21	Formic acid	CH	8.45
22	Trigonelline	C3′5H	8.82
22	Trigonelline	C1′H	9.11

**Table 2:** PLS-DA figures of merit for the discrimination between BC and control samples in the calibration and the two validation sets (LVs = 3).

Indices Test Validity	Calibration Set (BC vs. CTRL)	Validation Set (BC vs. CTRL)	Validation Set (BC vs. MONITOR + CTRL)
**True prevalence**	69.6%	73.3%	31.9%
**Sensitivity**	81.3% (68.1–89.8%)	86.4% (66.7–95.3%)	86.4% (66.7–95.3%)
**Specificity**	66.7% (45.4–82.8%)	87.5% (52.9–97.8%)	80.9% (67.5–89.6%)
**PPV ^a^**	84.8% (71.8–92.4%)	95.0% (76.4–9.1%)	67.9% (49.3–2.1%)
**NPV ^b^**	60.9% (40.8–77.8%)	70.0% (39.7–9.2%)	92.7% (80.6–97.5%)
**ACC ^c^**	76.8% (65.6–5.2%)	86.7% (70.3–4.7%)	82.6% (72.0–9.8%)
**PLR ^d^**	2.44 (1.31–4.53)	6.91 (1.10–43.54)	4.51 (2.45–8.3)
**NLR ^e^**	0.28 (0.15–0.52)	0.16 (0.05–0.45)	0.17 (0.06–0.49)

Note: ^a^ Positive predictive value; ^b^ Negative predictive value; ^c^ Diagnostic accuracy; ^d^ Positive Likelihood Ratio; ^e^ Negative Likelihood Ratio. Values in parentheses indicate the 95% confidence interval (CI).

**Table 3:** Identified metabolites and associated altered pathways in BC urines.

Altered Pathways in BC	Metabolites	*p* Value	Impact
Alanine, aspartate, and glutamate	Alanine, glutamine, succinate	6.5 × 10^−4^	0.26
Taurine and hypotaurine	Taurine, alanine	9.0 × 10^−3^	0.36
Aminoacyl-tRNA biosynthesis	Phenylalanine, glutamine, valine, alanine, lysine	1.6 × 10^−4^	0.56
Methane	Trimethylamine N-oxide, methanol	2.5 × 10^−2^	0.02
Arginine and proline	Citrate, succinate	9.0 × 10^−3^	0.08
Phenylalanine	Succinate, phenylalanine, hippuric acid	4.0 × 10^−3^	0.07
Nitrogen metabolism	Phenylalanine, taurine, glutamine	2.7 × 10^−3^	0.05

**Table 4:** Clinical and demographic data of patients included in this study.

Clinical data	Calibration Set	Validation Set
**Patients** (male/female)	24 (18/6)	7 (5/2)
**Age** (mean and standard deviation)	70 (11.15)	63 (5.13)
**Total samples**	69	84
**CTRL**	21	8
**MONITOR**	0	38
**NA**	0	16
**BC**	48	22
**Primary/Recurrent BC**	13/35	4/18
**Tumor stage** (Ta, T1, Tx)	33/13/2	17/4/1
**Tumor grade** (High/Low)	32/15 (UK:1)	20/1 (UK:1)
**Tumor size** (≥3/<3)	11/35 (UK:2)	7/15
**Tumor number** (1/2–7/≥8)	18/28/0 (UK:2)	14/8/0

Note: MONITOR samples from three patients included in the calibration set were included in the validation set. NA = non-available cystoscopic evaluation. UK = unknown.

## Supplementary Materials

The following are available online at https://www.mdpi.com/2072-6694/11/7/914/s1, Table S1: Confusion tables obtained from the evaluation of the predictive performance of PLS-DA models between pre-TUR (BC) and post-TUR or MONITOR samples (control) in the calibration (left) and validation (right) sets, Table S2: Patients and samples used in the calibration and validation set of PLS-DA model.
