# Supplementary material for: Urinary Metabolic Signatures Detect Recurrences in Non-Muscle Invasive Bladder Cancer
Source: Cancers (Basel). 2019 Jun 29;11(7):914. doi: 10.3390/cancers11070914 (PMC6678457; doi:10.3390/cancers11070914)
Supplement: Supplementary file 1 [file cancers-11-00914-s001.pdf]

cancers

MDPI

Supplementary Materials

Urinary metabolic signatures detect recurrences in non-muscle invasive bladder cancer

Alba Loras, M. Carmen Martínez-Bisbal, Guillermo Quintás, Salvador Gil, Ramón Martínez-Máñez and José Luis Ruiz-Cerdá

Table S1. Confusion tables obtained from the evaluation of the predictive performance of PLS-DA models between pre-TUR (BC) and post-TUR (CTRL) or MONITOR samples (control) in the calibration (left) and validation (right) sets.

Class	Calibration (CV)		External Validation		
	BC	CTRL	BC	CTRL	CTRL+ MONITOR
**Predicted as BC**	39	7	19	1	9
**Predicted as CTRL**	9	14	3	7	37

Table S2. Patients and samples used in the calibration and validation set of PLS-DA model.

Patient	Calibration		Validation		
	BC	CTRL	BC	CTRL	MONITOR
**1**	3	0	--	--	--
**2**	1	0	--	--	--
**3**	3	1	--	--	--
**4**	3	1	--	--	--
**5**	1	0	--	--	--
**6**	2	1	--	--	--
**7**	2	2	--	--	--
**8**	1	1	--	--	--
**9**	1	1	--	--	--
**10**	1	0	--	--	--
**11**	1	1	--	--	--
**12**	2	1	--	--	--
**13**	2	1	--	--	--
**14**	1	0	--	--	--
**15**	1	0	--	--	--
**16**	1	1	--	--	--
**17**	3	1	--	--	--
**18**	2	1	--	--	--
**19**	2	1	--	--	--
**20**	3	2	--	--	--
**21**	2	1	--	--	--
**22**	--	--	2	0	10
**23**	--	--	8	5	6
**24**	6	2	2	0	5
**25**	2	2	0	0	5
**26**	--	--	1	2	9
**27**	2	0	--	--	--
**28**	--	--	9	1	3

© 2019 by the authors. Licensee MDPI, Basel, Switzerland. This article is an open access article distributed under the terms and conditions of the Creative Commons Attribution (CC BY) license (http://creativecommons.org/licenses/by/4.0/).
